# Polish Experience of Implementing Vision Zero

**DOI:** 10.1007/s40719-017-0086-y

**Published:** 2017-04-22

**Authors:** Kazimierz Jamroz, Lech Michalski, Joanna Żukowska

**Affiliations:** Department of Highway and Transportation Engineering, Faculty of Civil and Environmental Engineering, Gdansk University of Technology, Gdańsk, Poland

**Keywords:** Road safety, Vision zero, Strategies, Road safety programming

## Abstract

**Purpose of Review:**

The aim of this study is to present an outline and the principles of Poland’s road safety strategic programming as it has developed over the last 25 years since the first Integrated Road Safety System with a strong focus on Sweden’s “Vision Zero”.

**Recent Findings:**

Countries that have successfully improved road safety have done so by following strategies centred around the idea that people are not infallible and will make mistakes. The human body can only take a limited amount of energy upon impact, so roads, vehicles and road safety programmes must be designed to address this.

**Summary:**

The article gives a summary of Poland’s experience of programming preventative measures that have “Vision Zero” as their basis. It evaluates the effectiveness of relevant programmes.

## Introduction

Poland’s soaring number of road deaths between the 1980s and 1990s^1^ was wrongly attributed to growing motorization and its inevitable effect on the level of road safety. Government bodies that were statutorily responsible for road safety used this as an excuse as they kept shifting the expenditure from road network improvements to other areas, which at the same seemed more important. And there was no reaction from the public to the growing traffic risks because after years of socialist economy, people were more than happy to accept the price of mobility, i.e. casualties and accident consequences, in return for the ability to drive. It was not until 1992 that a report by a group of World Bank experts made it absolutely clear that unless efforts are taken to change the system, accidents and casualties will grow [1]. What followed from this objective diagnosis were two decisions of the Polish government: in 1993, the prime minister established the National Road Safety Council and in 1994, the minister of transport commissioned the Scientific Research Committee to develop a research project called “An Integrated Road Safety Programme” which later came to be known as GAMBIT’96 [2]. Headed by the Gdansk University of Technology, the project was developed by a team of experts. Once complete, the programme was formally adopted by the government. That marked the start of its implementation at the regional level. In 2005, following Poland’s accession to the European Union, a new version of GAMBIT was developed to ensure that it fits in with European road transport safety goals and targets [3]. One of them was to adopt a systemic approach to safety programming and adapting the Swedish “Vision Zero”.

## “Vision Zero”: a Systemic Approach Toward Road Safety

The basic premise of “Vision Zero” in road transport is that serious road accidents (with fatalities or serious injuries) are unacceptable, and the long-term goal is to reduce their number to zero. From the work of its authors [4, 5••], it follows that Vision Zero is a:

- philosophy of planning traffic safety that assumes that no one should die or be seriously injured while using the road transport system,
- long-term action strategy consisting in the transformation of the road transport system to change the degree of responsibility between the road users and vehicles (including the motor industry) and the road and its environment (planners, designers and the road maintenance system; traffic control system, road emergency system and health care, government, parliament, etc.).

This approach, which assumes that there is nothing more valuable than human life, puts traffic safety above other criteria of assessing the functioning of the road transport system such as mobility, economy and environment. The basic requirement demanded of a road transport system is to provide mobility measured in the volume and speed or traffic. The volume of traffic impacts the number of accidents, whereas speed determines their consequences. The search for the reconciling of this conflict between mobility and safety led to many innovations and increasing investment in the development of road transport system safety. The search is heading in the direction of increasing the system of speed control and the use of ITS devices [6]. So the question arises whether the unquestionable ethical approach can beat the economic approach? Can the premises of Vision Zero be achieved, over how long and at what cost? What kinds of social economic and organisational barriers are there during its implementation? In spite of these misgivings, many countries, such as Norway [7•], Australia [6], Switzerland [8] and Iceland [9], adopted the new Swedish idea to improve traffic safety [10]. In recent years, there has been a growth of interest in Vision Zero in the USA [11]. Many American cities also want to eliminate road accident fatalities and in some cases serious injuries within the next 10 years [12].

## Implementing Vision Zero in Poland—“GAMBIT 2005”

Like many other countries, Poland also adopted “Vision Zero” as an ethically justified vision of traffic safety [13•]. The first programme to follow this vision in Poland was “National Traffic Safety Programme for the 2005–2013 (GAMBIT 2005)” [3]. The programme was created based on safe system approach and covered several important elements: diagnosis, vision, strategy, goals and priorities.

### Diagnosis

Each element of the safety programme is based on a diagnosis of the safety situation including an assessment of the existing traffic safety trends, assessment of the execution of previous traffic safety programmes, a description and assessment of the current situation as well as traffic safety forecasts. For example in 2004, which was construed as the basis for developing GAMBIT 2005, the primary Polish traffic safety indicators in terms of the number of fatalities were at a level they had been in Sweden, the Netherlands and England in the 1970s; two or three times as high as those today (e.g. the demographic rate was 106 casualties for 1 million population). The diagnostic analyses show that the groups at an especially high risk to die in traffic accidents in Poland in terms of the number of casualties and participation in traffic were unprotected road users (pedestrians, bikers, children) and young drivers. The primary problems of traffic safety in Poland are still the poor quality of the road infrastructure, lack of an efficient traffic safety management system and a poor traffic safety culture.

### Vision

**Fig. 1:**
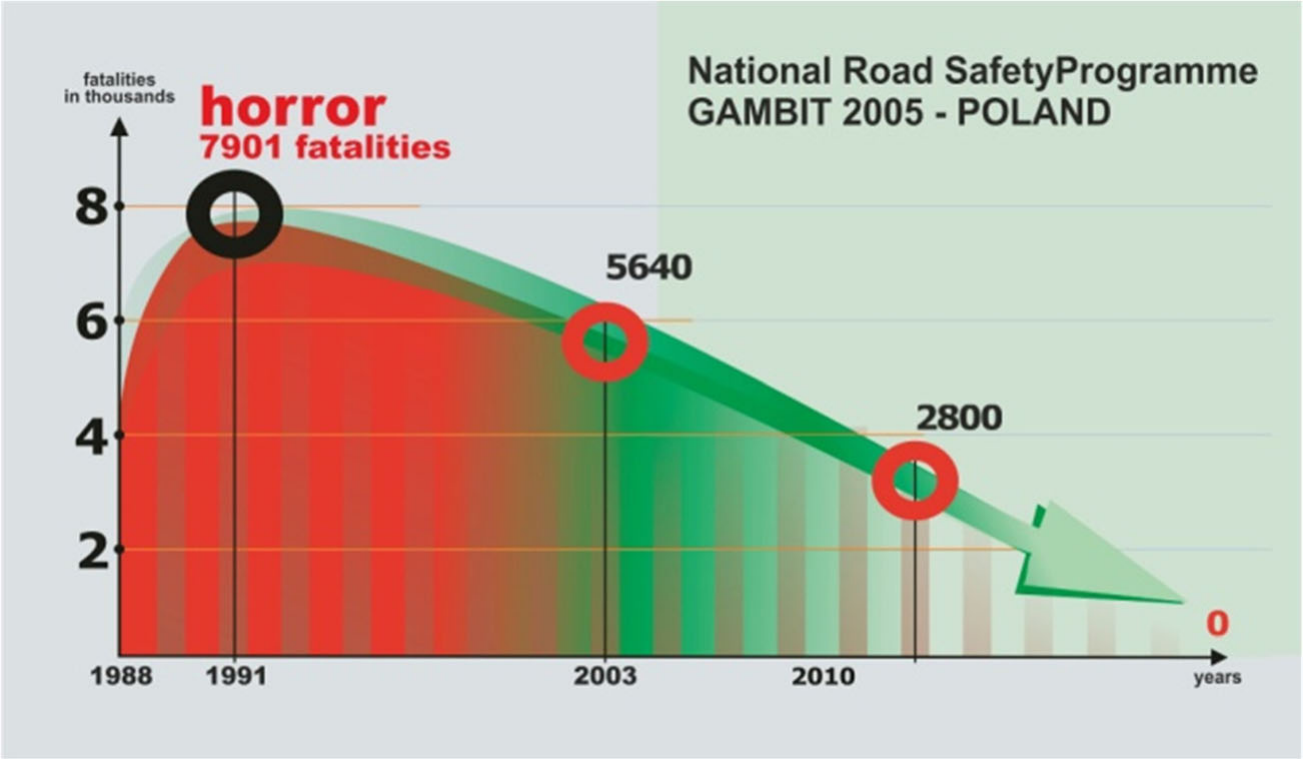
Strategic goal and road safety vision in Poland in the GAMBIT 2005 Programme [3]. Reprinted with permission from GAMBIT

The execution of an ambitious project or achieving a remote goal always begins with a vision. The assumptions of our programmes are similarly based on a clearly defined vision that points at an unambiguous direction and philosophy of our activity/programming. The state of traffic safety on the Polish road network at the time versus the leading countries in the European Union (United Kingdom, Netherlands, Sweden, Germany) meant that the measures to improve traffic safety had to be treated as one of the most important priorities in the development and maintenance of the national road network in Poland. It was assumed that the long-term ethically justified vision of traffic safety was to completely eliminate fatalities on Polish roads (Fig. 1) [3].

### Strategy

The traffic safety strategy document is an instrument that expresses a joint approach to solving the problem of traffic safety and a guide that helps to achieve the outlined goals. The Strategy document accepted by the stakeholders is a result of a process that strongly involves all of these stakeholders, especially in determining the strategic goal and the selection of priorities. This means reaching a compromise between expectations and the capability of meeting them. Strategy is one of the basic instruments of modern management, including road network management.

In the GAMBIT 2005 Programme, the strategic goal was to reduce by 2013 the number of fatalities by over 50% versus 2003, i.e. no more than 2800 fatalities [3]. Moreover, two interim stage goals were set, which were milestones for the implementation of the strategy:

- 2007—no more than 4300 fatalities,
- 2010—no more than 3500 fatalities.

### Detailed Goals and Priorities

Achieving such an ambition strategic goal requires undertaking many comprehensive, intensive, efficient and integrated measures in the entire country. However, it is impossible to carry out all these measure simultaneously because it requires investing a lot of work and resources, while the results are not always proportionate to the effort. Considering the most important traffic safety problems and the groups at risk of being traffic accident casualties within the strategic timeframe, 5 detailed strategic goals were adopted by 2013, which were divided into 15 priorities, with each priority having from several to over a dozen measures. The detailed goals included:

1. Creating bases to carry out effective and long-term traffic safety measures (organisational structures, traffic safety management, sectoral measures),
2. Modifying road users’ hazardous behaviour (speed, seat belts, alcohol),
3. Protecting pedestrians, children and bikers,
4. Streamline the most dangerous spots and road sections (inspections, safe roads, modern traffic management),
5. Reducing the frequency of accidents (traffic safety on board vehicles, safe road environment, optimising the emergency rescue system).

A systemic approach to solving traffic safety problems was applied in most of the priorities, assuming taking measures in several sectors at once (e.g. education, traffic monitoring, engineering and emergency rescue) because it was found that such an approach will yield much better results than a sum of piecemeal measures. A systemic approach to solving traffic safety issues requires a transparent traffic safety management system project, adopting the appropriate legal, organisational and financial forms, determining how the organisations, institutions and individuals involved in the traffic safety improvement process work together.

## Recommendations for the Health Sector

The health service also plays a vital role in achieving “Vision Zero” in Poland. The results of saving the lives of traffic accident casualties depend on the optimising of rescue measures in line with the “chain of survival”, which consists of early notifying of emergency medical rescue teams, early providing of aid by professional emergency rescue services and early commencement of treatment in professional emergency wards. Therefore, it is recommended to take measures to improve its effectiveness:

1. At the strategic level by employing social health change theories in health protection programmes, which can change the behaviour of individual road users and entire social groups toward more pro-health conduct, the manner in which legislators and those who implement engineering solutions as a way to protect society from hazards to life and health make their decisions; organising top-notch systems of road emergency rescue, health protection and for providing aid to the victims of traffic accidents.
2. At the tactical level by:
  Shortening the time of detection and notifying about traffic incidents (supporting the creation of public-safety answering points based on the 112 emergency number and the development of ITS in terms of the automatic detection of traffic incidents),
  Shortening the travel time to the site of the accident and the time it takes to transport the casualties to the nearest emergency hospital by supporting measures to optimise putting emergency units in powiats (counties) and supporting the development of the Air Ambulance Service,
  Spreading the standardising of action at the site of the accident (implementing common rescue procedures shared by all emergency services, supporting joint drills with all the emergency services, monitoring the quality of the work of the emergency rescue services),
  Developing specialised medical units (supporting the creation of Emergency Rooms, and training medical rescue workers).
3. At the operational level by promoting individual health-oriented models (belief in health, justified action) that cause intentions, subjective standards and attitudes to gradually change from a lack of interest in health to pro-health behaviours aiming at the protection of life and health in traffic, learning rescue skills by taking part in training courses to allow quick rescue operations at the site of the accident while waiting for the emergency rescue services to arrive.

## Effectiveness of Vision Zero in Poland

**Fig. 2:**
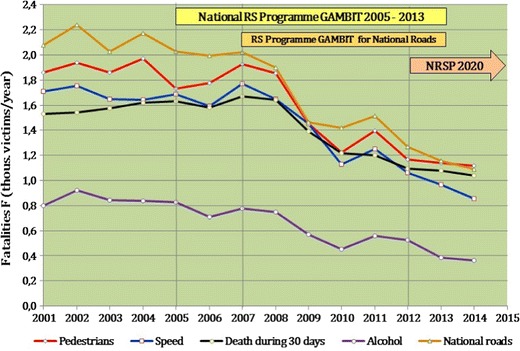
Change in the number of traffic accident fatalities in Poland in 2001–2015 by traffic safety problems and the implementation periods of traffic safety programmes. Reprinted with permission from Gambit

In 2012, an interim assessment up to 2010 was performed on the GAMBIT 2005 programme [14]. The interim goal for 2010 was no more than 3500 fatalities, whereas 3907 persons died on Polish roads, i.e. the number of fatalities fell by 31% instead of the target 38%. At the initial stage of the implementation of the GAMBIT 2005 (2005–2008), there was stagnation or a slight drop in the number of fatalities. In spite of a large drop in the number of fatalities in 2008–2010 (Fig. 2), the interim target of reducing fatalities was carried out in 80%.

In 2011, there was a disturbance in this downward trend: the number of traffic accident fatalities rose year-on-year by 7%, whereas in 2012, the downward trend returned and lasted until the end of 2015. There was a large reduction in the number of fatalities in 2003–2010 in terms of driving under the influence of alcohol and collisions with trees (46–45%) which means that there were a great deal of effective measures taken to solve these problems. The smallest reduction of fatalities (23%) was achieved for individuals who died within 30 days of the accident, which shows that traffic accidents in Poland tend to be very serious and the inadequacies of the health care system.

Over the analysed period of the implementation of the GAMBIT 2005 programme, there were many national educational, preventive and infrastructural measures taken in harmony with the programme. At the same time, in many cases, there were political and administrative decisions made that were inconsistent with the programme. Unfortunately, only 84 out of 144 tasks meant for realisation were carried out (58%). Although the measures taken improved traffic safety. It is estimated that as a result of the Programme measures carried out over the 7 years of its implementation:

- ca. 6000 lives were saved from death in road accidents,
- the savings due to this amounted to ca. 34.5 billion PLN.^2^

The most effective measures were:

- developing and implementing Traffic Safety Programmes at the voivodeship (regional) and powiat (county) levels, covering over a dozen voivodeships, cities and powiats,
- developing and implementing a sectoral set of programmes (for national roads administration, the police),
- beginning the construction of the Polish Traffic Safety Observatory and setting up two regional observatories,
- changes in the ways drivers are trained and examined,
- implementing and developing a monitoring system (speed control, control of drivers’ working hours),
- standardising regulations for bikers,
- intensive construction of express roads and motorways, building safe junctions, using traffic calming measures,
- introducing traffic safety audits,
- upgrading the emergency rescue and post-accident protection systems.

Some of the undertaken measures did not yield the expected results, were poorly carried out or, regrettably, many important measures planned in the traffic safety programme were not implemented, including:

- failure to designate the leader of the GAMBIT’2005 Programme,
- failure to streamline the structures of the institutions dealing with national traffic safety, especially the National Traffic Safety Council (lead agency),
- failure to establish local executive actors (inspectors, officers, leaders),
- failure to implement an efficient system of funding traffic safety measures,
- failure to implement an efficient monitoring of the progress of the strategy’s implementation,
- failure to streamline the promotion of effective measures for traffic safety.

The seriousness of traffic accidents in Poland (10 fatalities per 100 accidents), issues with the emergency rescue system and the problems of the Polish health service still cause over 30% accident casualties to die within 30 days of the instance of the accident. This share has not dropped, as was expected in the GAMBIT 2005 (12.7% in 2013), but has remained close to the baseline (28% in 2003). For this reason, it is necessary to intensify action in three directions: reducing the seriousness of accidents (through infrastructural, organisational and management measures), streamlining the traffic emergency rescue system and post-accident aid for the casualties of accidents.

Based on an assessment, the goals and measures were verified and a new National Traffic Safety 2020 Programme (NRSP 2020) (Fig. 2) [15] was developed with the guidelines of the UN Decade of Action for Road Safety [16] and EU recommendations [17].

## When the “Zero Fatalities” May Be Achieved in Poland?

**Fig. 3:**
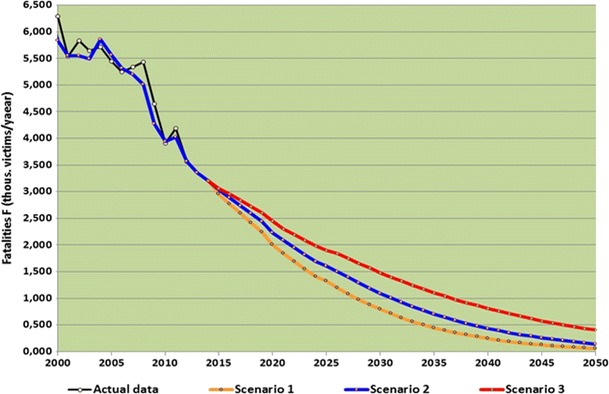
Forecast of fatalities in Poland until 2050 for different development scenarios. Reprinted with permission. [22•]

An analysis of the possibility of achieving Vision Zero in Poland was carried out on the basis of theoretical analyses with the aid of simulations. Based on the studied literature [18, 19, 20] and own research results [21], a concept of changing traffic safety in Poland measured by road fatality rate was adopted. According to this concept, the level of traffic safety in a country changes in a non-linear way depending on changes in social and economic development measured by the gross domestic product per inhabitant. For the purpose of the analysis, long-term forecasting of the number of road fatalities were used [22•, 23]. Using the developed forecasting models, the forecast of the number of fatalities for three scenarios of the development of the traffic safety system in Poland until 2050 were made. These scenarios differed in the population in 2050, gross domestic product and traffic safety measures to be implemented. As a result of these calculations, an estimate of the number of fatalities in 2015–2050 was made for the scenarios presented in Fig. 3. The results presented in the figure show that Poland’s expected further social and economic growth and the resulting changes of other independent variables (according to the current trends in countries with the best traffic safety) will lead to a consistent reduction in the number of traffic accident fatalities. However, these changes may not be enough to achieve “Vision Zero” for traffic accident fatalities in 2050 (Fig. 3) [22•].

## Conclusions

In spite of many measures and interventions taken to improve traffic safety, Poland is still among the EU countries with the highest risk to life and health in traffic. It is estimated that, with this state of affairs, over 100,000 people may die in traffic accidents by 2050, with over 1,000,000 injured. The material and social losses caused by these traffic incidents may cost as much as 1 billion USD. Therefore, all measures to protect the lives and health of road users should be a priority. Developing and implementing further national, sectoral, regional and local traffic safety programmes will lead to a consistent reduction in the number of fatalities in Poland, but the results still remain far from the expectations.

An analysis of the possibility of achieving “Vision Zero” in Poland 2050 indicates that this strategic goal can be achieved by using additional and intensified strategic measures and interventions. Among the analysed interventions, high effectiveness and efficiency in the reduction of the number of fatalities can be achieved by measures directed at changing the behaviour of road users (driving at safe speeds, no driving under the influence of alcohol, using safety devices in vehicles), protecting pedestrians and bikers, developing safe and modern road infrastructure (roads with high safety standards, forgiving roads) and also at changing the way the country operates (developing the traffic safety management system, developing the health protection system). The consistent and intensive implementation of the proposed measures and use of new technology will make it possible to get very close to “Vision Zero” in 2050.

## Footnotes

1 In 1989, Poland transitioned from its previous political system to one that promoted free market economy and civil society.

2 1PLN equals 4USD
